# A Systematic Study of the Mechanism of Acacetin Against Sepsis Based on Network Pharmacology and Experimental Validation

**DOI:** 10.3389/fphar.2021.683645

**Published:** 2021-08-16

**Authors:** Yuanshuo Ouyang, Yi Rong, Yanming Wang, Yanli Guo, Liya Shan, Xiushi Yu, Li Li, Junqiang Si, Xinzhi Li, Ketao Ma

**Affiliations:** ^1^ Key Laboratory of Xinjiang Endemic and Ethnic Diseases, Ministry of Education, Shihezi University School of Medicine, Shihezi, China; ^2^ NHC Key Laboratory of Prevention and Treatment of Central Asia High Incidence Diseases, First Affiliated Hospital, Shihezi University School of Medicine, Shihezi, China; ^3^ Department of Physiology, Shihezi University School of Medicine, Shihezi, China; ^4^ Department of Pathophysiology, Shihezi University School of Medicine, Shihezi, China

**Keywords:** sepsis, acacetin, network pharmacology, molecular docking, macrophage

## Abstract

Sepsis is a dysregulated systemic response to infection, and no effective treatment options are available. Acacetin is a natural flavonoid found in various plants, including *Sparganii rhizoma*, *Sargentodoxa cuneata* and *Patrinia scabiosifolia*. Studies have revealed that acacetin potentially exerts anti-inflammatory and antioxidative effects on sepsis. In this study, we investigated the potential protective effect of acacetin on sepsis and revealed the underlying mechanisms using a network pharmacology approach coupled with experimental validation and molecular docking. First, we found that acacetin significantly suppressed pathological damage and pro-inflammatory cytokine expression in mice with LPS-induced fulminant hepatic failure and acute lung injury, and *in vitro* experiments further confirmed that acacetin attenuated LPS-induced M1 polarization. Then, network pharmacology screening revealed EGFR, PTGS2, SRC and ESR1 as the top four overlapping targets in a PPI network, and GO and KEGG analyses revealed the top 20 enriched biological processes and signalling pathways associated with the therapeutic effects of acacetin on sepsis. Further network pharmacological analysis indicated that gap junctions may be highly involved in the protective effects of acacetin on sepsis. Finally, molecular docking verified that acacetin bound to the active sites of the four targets predicted by network pharmacology, and *in vitro* experiments further confirmed that acacetin significantly inhibited the upregulation of p-src induced by LPS and attenuated LPS-induced M1 polarization through gap junctions. Taken together, our results indicate that acacetin may protect against sepsis *via* a mechanism involving multiple targets and pathways and that gap junctions may be highly involved in this process.

## Introduction

Sepsis is a severe systemic inflammatory response to microbial infection, and lipopolysaccharide (LPS) is well known to induce this phenomenon (Chen et al., 2020). D-Galactosamine (GalN)-sensitized mice administered LPS and mice administered only LPS by intratracheal instillation are recognized as promising experimental models of fulminant hepatic failure (FHF) and acute lung injury (ALI) (Zhang et al., 2019; Liu et al., 2020a). Macrophage polarization is involved in the pathogenesis of sepsis, an uncontrolled inflammatory response caused by infection or acute insult (Fan et al., 2020). Excessive M1 polarization may lead to exacerbated inflammatory reactions and initiate injury in the host.

Acacetin (5,7-dihydroxy-40-methoxyflavone), an O-methylated flavone monomer, is a bioactive constituent found in various plants, including *Sparganii rhizoma, Sargentodoxa cuneata* and *Patrinia scabiosifolia* (Hu et al., 2020; Lu et al., 2021). Acacetin has diverse biological functions, including anti-inflammatory, antioxidant, and anti-microbial activity, and exerts protective effects on cardiac tissue and neurons (Singh et al., 2020). Various studies have reported the anti-inflammatory activity of acacetin *in vitro* and *in vivo*. For example, acacetin was shown to attenuate ALI in LPS-induced mice *via* augmenting haem oxygenase-1 (HO-1) activity (Sun et al., 2018) and to protect against GalN/LPS-induced liver injury by suppressing the TLR4 signalling pathway (Cho et al., 2014). Moreover, acacetin strongly inhibited the expression of the pro-inflammatory cytokines inducible nitric oxide synthase (iNOS) and COX-2 in LPS-activated RAW264.7 cells and in a mouse model of tetradecanoyl phorbal acetate (TPA)-induced tumours (Pan et al., 2006). These results suggest that acacetin ameliorates sepsis by inhibiting inflammation and promoting redox homeostasis, but the underlying molecular mechanism remains unclear.

Network pharmacology is an effective method for screening drug-disease targets and for predicting the possible underlying mechanisms *via* bioinformatics analyses (Li et al., 2020; Xia et al., 2020; Zhang et al., 2021). For example, acacetin was shown to be a bioactive compound in *Sparganii rhizoma*, which has anti-gastric cancer activity as determined by network pharmacology analysis (Lu et al., 2021). Acacetin is also the active ingredient in *Sargentodoxa cuneata* and *Patrinia scabiosifolia,* which improve pelvic inflammatory diseases related to dampness-heat stasis syndrome as determined by network pharmacology analysis (Hu et al., 2020). In this study, we investigated the potential targets of acacetin in subjects with sepsis and predicted the underlying mechanisms by network pharmacology coupled with molecular docking and experimental validation.

## Materials and Methods

### Reagents

Acacetin (MedChemExpress, molecular weight: 284.26 g/mol), LPS (Sigma, 055:B5, L2880), and Gap27 (MedChemExpress) were used in the experiments.

### Animals and Model Treatment

Healthy male C57BL/6 mice (8–12 weeks old, 24–26 g) were purchased from the Animal Experimental Centre of the Fourth Military Medical University, China. All mice were bred in a specific pathogen-free animal facility at the Department of Biochemistry and Molecular Biology, School of Basic Medical Science, Xi’an Jiaotong University Health Science Center. All animal experiments were approved by the Institutional Animal Ethics Committee of Xi’an Jiaotong University (no. 2017–666). All animal experiments were performed in accordance with standard animal research guidelines. C57BL/6 mice were randomly assigned to one of six groups (*n* = 5): the control group, acacetin treatment group, LPS-induced ALI model group, LPS + acacetin treatment group, LPS/GalN-induced FHF model group and LPS/GalN + acacetin treatment group. ALI was induced by the intratracheal injection of LPS (5 mg/kg) (Zhang et al., 2019), and FHF was induced by the intraperitoneal injection of GalN (800 mg/kg) and LPS (40 μg/kg) (Liu et al., 2020a). An equivalent amount of PBS was administered to mice in the control group. Two hours before the intraperitoneal injection of acacetin (50 mg/kg) to the solvent comparison and treatment groups, acacetin was dissolved in 5% core oil. The mice were sacrificed at 10 h after ALI modelling and at 6 h after FHF modelling.

### Histology

The lung and liver tissues were collected and fixed in a 4% paraformaldehyde solution for 48 h. The tissues were dehydrated, embedded in paraffin, and sliced into 5 μm-thick sections. The sections were stained with haematoxylin and eosin (H & E) reagent and visualized under a light microscope.

### ELISA

To assess the effects of acacetin on pro-inflammatory mediators, appropriate ELISA kits (Jioin, Shanghai, China) were used. The levels of the pro-inflammatory mediators TNF-α, IL-6, and IL-1β in lung and liver homogenates from LPS-induced mice were measured.

### Cell Culture

RAW264.7 macrophages were obtained from academician Rikard Holmdahl (Sweden Medical Inflammation Research of the Karolinska Institute). The cells were cultured in a thermostatic incubator at 37°C and 5% CO_2_. This cell line, used to screen for molecular mechanisms, was cultured in DMEM (Gibco, Thermo Fisher Scientific, Inc.) supplemented with 10% FBS (Gibco, Carlsbad, CA, United States) and 1% streptomycin/penicillin. The cells were passaged when they reached 70–80% confluence.

### MTT Assay

RAW264.7 cells were cultured in a 96-well plate at 5 × 10^3^ cells/well. Cells in the treatment groups were administered different amounts of acacetin (1, 5, and 10 μMol) dissolved in DMSO, while those in the model group were administered LPS (100 ng/ml), and those in the control and blank (no cells) groups were administered an equivalent amount of medium before growth for an additional 24 h. Then, MTT solution was added and continuously incubated at 37°C for 4 h. The medium in each well was discarded, after which 150 μl of DMSO was added, and the cells were shocked on a microvibrator for 5 min. The optical density at 490 nm was determined with a plate reader (VICTOR X5, PerkinElmer, MA, United States).

### Flow Cytometry

The experimental groups were divided into a control group, an LPS model group, and treatment groups that were administered different amounts of acacetin (1, 5, 10 μMol). Groups other than the control and LPS model groups were pre-treated with acacetin for 4 h and then stimulated with LPS (100 ng/ml) for 24 h. Flow cytometry was performed on single-cell suspensions of RAW264.7 macrophages. For intracellular staining, cells were fixed and permeabilized using BD Cytofix/Cytoperm solution (BD Biosciences, NJ, United States). The following monoclonal antibodies were used: the M1-type surface markers CD86-PE (1:150; BD Biosciences, NJ, United States) and iNOS-PEcy7 (1:150; BD Biosciences, NJ, United States). Samples were measured by flow cytometry (Guava, Millipore), and the data were analysed using Flow Jo software (TreeStar Inc., OR, United States).

### Western Blotting

The cell grouping, stimulation, and culture were carried out as described for the flow cytometry experiment. The cells were harvested and lysed with radioimmunoprecipitation assay (RIPA) buffer and phenyl methyl sulfonyl fluoride (PMSF), and the protein content was confirmed by the BCA method. Equivalent amounts of protein from each sample were loaded onto 10% SDS-PAGE gels and then transferred onto PVDF membranes (EMD Millipore, CA, United States). After being blocked with 10% skimmed milk for 2 h, the blots were probed with the following primary antibodies: CD86 (1:1,000; cat. no. ab53004; Abcam), iNOS (1:800; cat. no. ab15323; Abcam), p65 (1:1,000; cat. no. ab16502; Abcam), p-p65 (1:1,000; cat. no. ab86299; Abcam), Src (1:1,000; cat. no. 2109T; CST), p-Src (1:1,000; cat. no. 6943T; CST), Cx43 (1:1,000; cat. no. AP1541b; Abcepta) and GAPDH (1:2,000; cat. no. bs-2188R; BIOSS). The cells were gently incubated overnight at 4 °C and subsequently incubated with the corresponding secondary antibodies diluted 1:5,000 with 5% skimmed milk. The membranes were washed with TBST, incubated with ECL reagent (GE Healthcare Life Sciences, United Kingdom) and developed. Quantity One software (Bio-Rad, Hercules, CA, United States) was used to analyse the collected images.

### Quantitative RT-PCR

The cell culture and stimulation were performed as described for Western blot analysis. Only one acacetin (10 μmol) treatment group was used. After stimulation, the cells were harvested with TRIzol reagent (Life Technologies, CA, United States), and total RNA was extracted and reverse transcribed into cDNA using a ReverTraAce RT kit (Thermo Fisher, MA, United States). Real-time quantitative PCR was carried out using SYBR Green Master Mix (Roche, Germany) according to the manufacturer’s protocols. The following gene primer sequences were obtained from Shanghai Gene Pharma: CD86, 5′-ACG​GAG​TCA​ATG​AAG​ATT​TCC​T-3′ (forward), 5′-GAT​TCG​GCT​TCT​TGT​GAC-ATA​C-3′ (reverse); iNOS, 5′-GTT​TAC​CAT​GAG​GCT​GAA​ATC​C-3′ (forward), 5′-CCT​CTT​GTC​TTT-GAC​CCA​GTA​G-3′ (reverse); and β-actin, 5′-CAC​GAT​GGA​GGG​GCC​GGA​CTC​ATC-3′ (forward), 5′-TAA​AGA​CCT​CTA​TGC​CAA​CAC​AGT-3′ (reverse). mRNA abundance was quantified using the Ct values obtained by 2^−ΔΔ^Ct comparative method.

### Network Pharmacology Approach

The acacetin chemical formula was imported into the Swiss Target Prediction database (http://swisstargetprediction.ch/) for drug-target prediction analysis, and the keyword “sepsis” was then searched in the Gene Cards database (https://www.genecards.org/) to identify disease targets. The overlapping targets among compound and sepsis targets were used to draw a Venn diagram.

The protein-protein interaction (PPI) network was obtained by introducing these potential overlapping targets of acacetin and sepsis into the STRING database (https://string-db.org) with the species limited to “*Homo sapiens*” and a confidence score >0.9. The results were further visualized by Cytoscape 3.7.0. The degree of freedom was reflected by the node size and colour. Subsequently, the top four key targets were identified as hub targets based on their degree, centrality and proximity to hub proteins.

The hub targets were converted into their Ensembl gene names (www.ensembl.org/biomart/), and the OmicShare server (http://www.omicshare.com/) was then used for the analysis. The converted Ensembl gene names were used as input, and Gene Ontology (GO) enrichment and Kyoto Encyclopaedia of Genes and Genomes (KEGG) enrichment analyses were performed. The molecular function (MF), biological process (BP) and cellular component (CC) terms enriched in the targets were determined, and pathway enrichment of the top 20 pathways was visualized with the R package.

These results were summarized and loaded into Cytoscape 3.7.0 to construct the acacetin-target-GO-KEGG-sepsis network. In the network, edges represent interactions between nodes, and nodes represent drugs, diseases, BPs, pathways and targets.

### Molecular Docking

The 3D crystal structures of ESR1 (PDB code: 5ACC), COX-2 (gene: PTGS2, PDB code: 4RRX), SRC (PDB code: 4U5J) and EGFR (PDB code: 6JZ0) were obtained from the Protein Data Bank (PDB). The 2D structure of acacetin was downloaded from the ZINC15 database (https://zinc15docking.org/). The original ligand active site residues were identified from the PDB coordinates and recorded. The protein structures were prepared by removing all nonreceptor atoms, including water, ions, and miscellaneous compounds, using Discover Studio Visualizer. Docking experiments were performed using CDOCKER with receptor-ligand interactions. The -CDOCKER_INTERACTION_ENERGY value was determined to evaluate bonding. The binding results were visualized as 3D and 2D diagrams using Discovery Studio Visualization version 4.5 (Accelrys, Inc., San Diego, CA 92121, CA, United States).

### Statistical Analyses

Statistical evaluations were performed using the GraphPad Prism 5 package (GraphPad Software Inc., San Diego, CA, United States). Multiple comparisons were performed by one-way ANOVA followed by Tukey’s post hoc significant difference test. *p* values less than 0.05 indicate statistical significance.

## Results

### Protective Effect of Acacetin Against Sepsis *in vivo*

**FIGURE 1:**
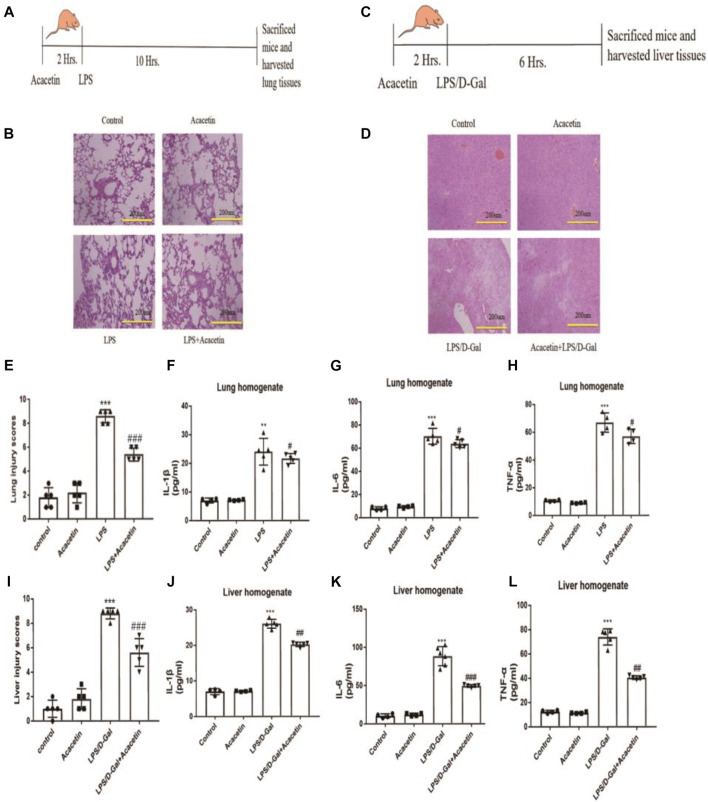
The protective effect of acacetin against sepsis *in vivo*. **(A)** and **(C)** Methods to generate two inflammatory models and diagram showing the drawing time. **(B)** and **(D)** Histopathological damage to the lung (× 200) and liver (× 200). **(E)** and **(I)** Lung and liver injury scores. The lung **(F)** and liver **(J)** homogenate levels of IL-1β **(G)**, **(K)** IL-6 **(H)**, and **(L)** TNF-α were determined by ELISA. The data are expressed as the mean ± SEM (n = 5). *p < 0.05, **p < 0.01, ***p < 0.001, compared with the control group; ^#^ *p* < 0.05, ^##^ *p* < 0.01, ^###^ *p* < 0.001, compared with the LPS-stimulated group.

To assess the protective effect of acacetin against sepsis, we utilized LPS-induced FHF and ALI models. Compared with the control group, the FHF model group showed congestion, oedema, inflammatory cell infiltration, and a broadened pulmonary interval (*p* < 0.05), and the ALI model group showed more inflammatory areas in the portal vein and more necrotic hepatocytes (*p* < 0.05). Compared with the FHF and ALI model groups, the acacetin group showed less severe lung and liver injury symptoms as determined by HE staining (Figures 1B,D), and the injury scores of the acacetin group were significantly lower than those of the model group (Figures 1E,I; *p*<0.05). The levels of IL-1β, IL-6 and TNF-α in the liver and lung tissues of the model group were significantly higher than those in the control group tissues, while those in the acacetin group were decreased compared to those in the control group (Figures 1F–H,J–L *p* < 0.05). These results indicated that acacetin improves liver and lung injury by decreasing the levels of IL-1β, IL-6 and TNF-α.

### Protective Effect of Acacetin Against Sepsis *in vitro*

**FIGURE 2:**
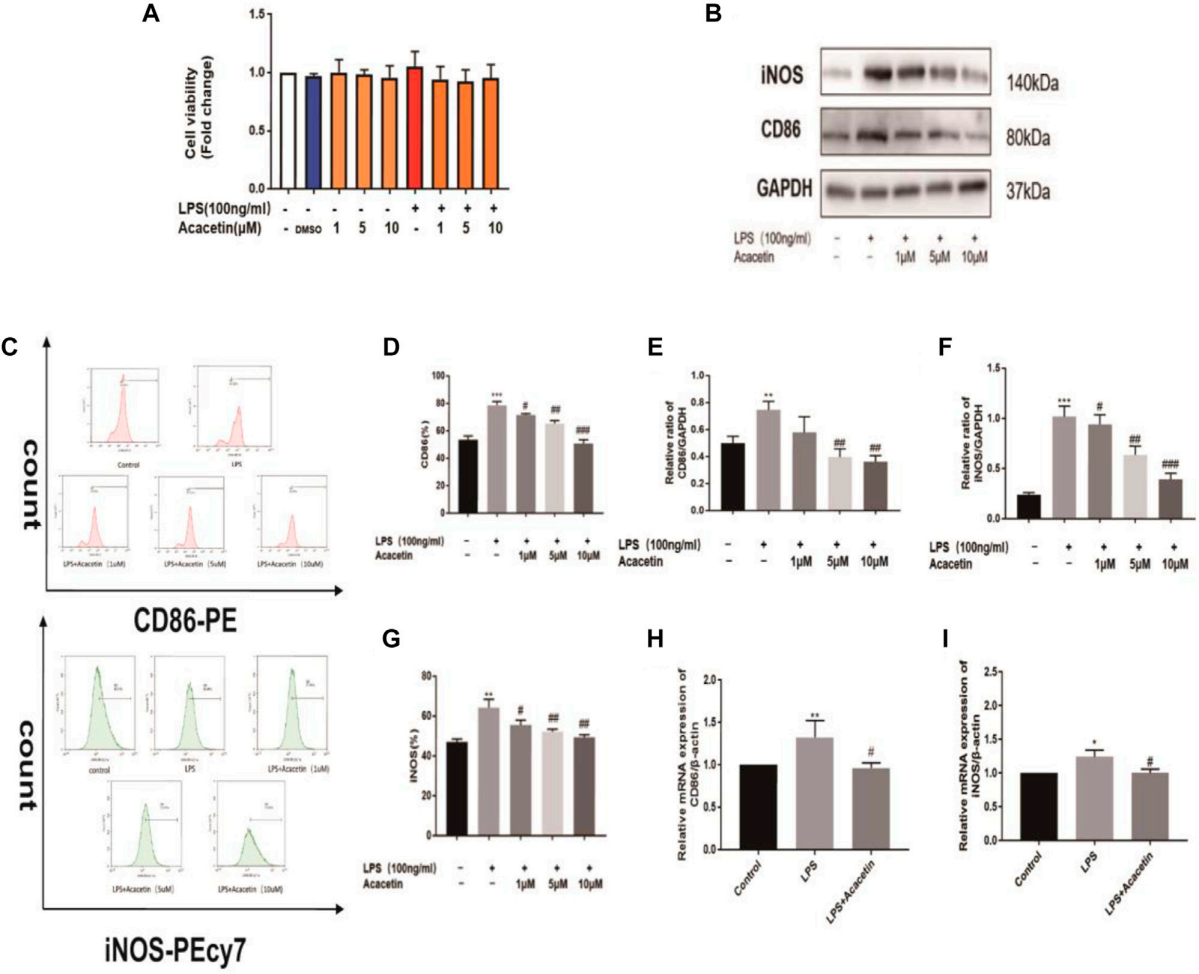
The protective effect of acacetin against sepsis *in vitro*. **(A)** The effect of acacetin on cell viability as determined by the MTT assay. The protein levels **(B, E, F)**, positive cells identified by FACS **(C, D, G)**, and mRNA levels **(H, I)** of the M1 polarization markers CD86 and inducible nitric oxide synthase (iNOS) in RAW264.7 cells. The data are expressed as the mean ± SEM (*n* = 3). *p < 0.05, **p < 0.01, and ***p < 0.001 compared with the control group; ^#^ *p* < 0.05, ^##^ *p* < 0.01, ^###^ *p* < 0.001 compared with the LPS-stimulated group.

Macrophage polarization is involved in the pathogenesis of sepsis. To assess the protective effect of acacetin on sepsis and the underlying mechanism *in vitro*, we observed its effect on M1 macrophage polarization induced by LPS in RAW264.7 cells. As shown in Figure 2A, treatment with 1, 5, and 10 μMol acacetin had no toxic effects, and the agent was thus used at these nontoxic concentrations in further experiments. Compared with those in the control group, the CD86 and iNOS protein levels, number of positive cells determined by flow cytometry, and mRNA levels were increased in the LPS-stimulated group. Compared with LPS, acacetin attenuated the CD86 and iNOS protein levels (Figures 2B,E,F), the number of positive cells determined by fluorescence-activated cell sorting (FACS) (Figures 2C,D,G) and the mRNA levels (Figures 2H,I). Additionally, exposure to various doses of acacetin (1, 5, and 10 μMol) attenuated the expression of CD86 and iNOS in M1-type cells in a concentration-dependent manner (*p* < 0.05). These phenomena revealed that acacetin helped to quickly reduce the CD86 and iNOS levels in M1-type macrophages.

### Elucidating the Mechanisms Underlying the Effects of Acacetin on Sepsis *via* a Network Pharmacology Approach

**FIGURE 3:**
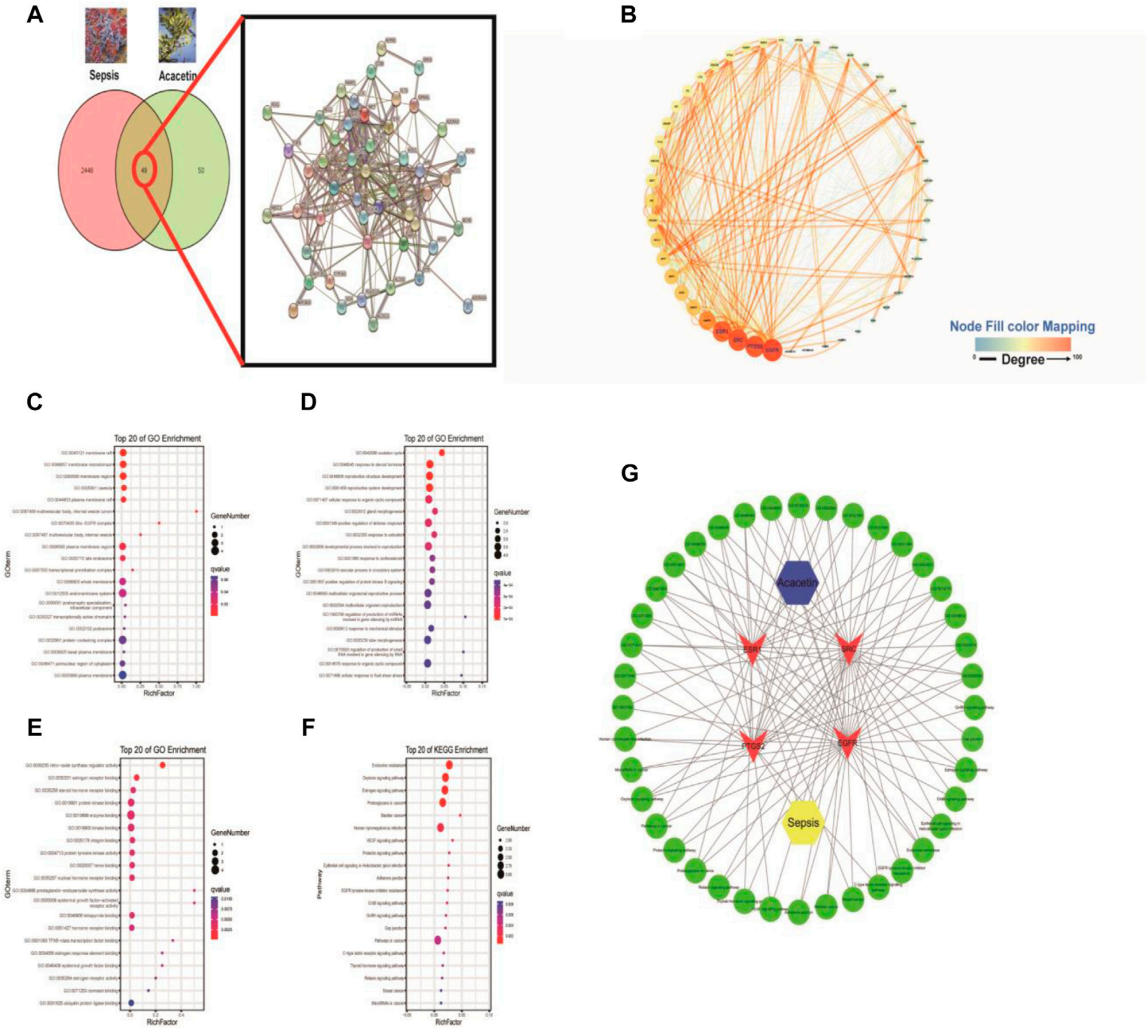
The underlying mechanisms by which acacetin affects sepsis as determined by a network pharmacology approach. **(A)** All of the targets of acacetin and sepsis and the 49 intersecting targets of acacetin and sepsis were identified *via* a Venn diagram and PPI network. **(B)** The top four hub genes/targets of both acacetin and sepsis were identified and arranged according to their degree value with an algorithm using Cytoscape software. **(C–F)** With the OmicShare service R-language packages, the four core targets were subjected to GO analyses of cellular component (CC), biological process (BP), and molecular function (MF) terms and KEGG pathway analysis. The bubble diagram shows the top 20 terms related to the activity of acacetin against sepsis. **(G)** Network constructed from the bioinformatics analysis results highlighting the detailed interactions between acacetin-targets-GO-KEGG-sepsis. Hub targets are represented by a red V, and the top 20 BP terms and pathways involved in the activity of acacetin against sepsis are shown in green.

**FIGURE 4:**
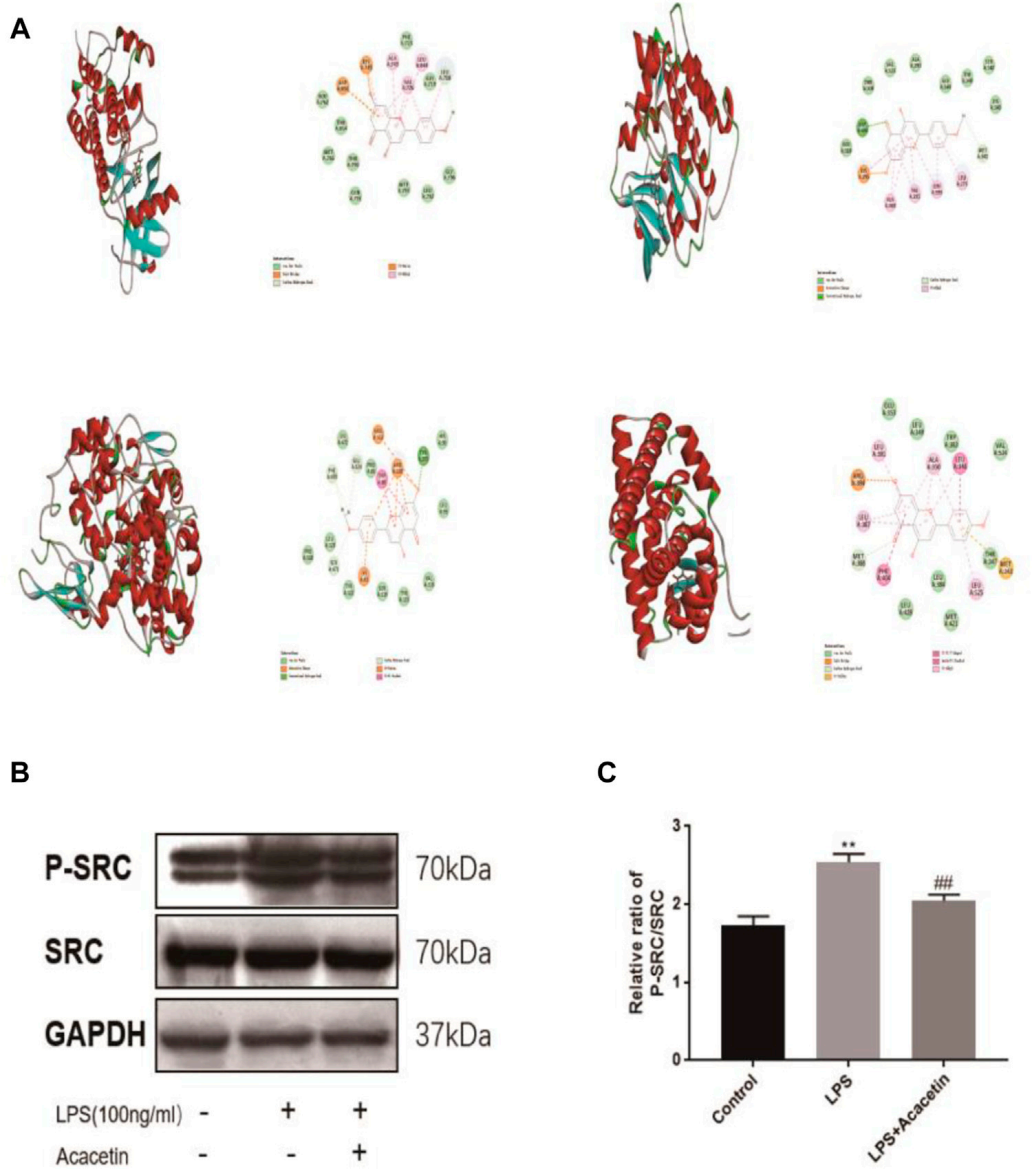
Molecular docking and experimental validation of acacetin binding core targets predicted by network pharmacology. **(A)** EGFR, SRC, PTGS2 and ESR1. All pictures show the 3D docking of ligands in the active binding pocket, with the hydrophobic effect area and the 2D interaction patterns between the ligands and proteins and the important interactions between the ligand atoms and amino acid residues of the proteins being displayed. **(B–C)** The expression levels of Src and phosphorylated Src were assessed via Western blotting. The data are expressed as the mean ± SEM (*n* = 3). *p < 0.05, **p < 0.01, and ***p < 0.001 compared with the control group; ^#^ *p* < 0.05, ^##^ *p* < 0.01, ^###^ *p* < 0.001 compared with the LPS-stimulated group.

The canonical SMILES string for acacetin was input into the Swiss Target Prediction database to identify the targets of acacetin, and a total of 99 potential drug targets were found. There were 2,497 potential targets of sepsis in the GeneCards database. As shown in Figures 3A,4 common targets were included in the acacetin-sepsis target Venn diagram. The 49 intersectional targets were imported into the STRING database to explore the relationships among these targets (Figure 3A). A PPI network of the 49 common targets between acacetin and sepsis was constructed based on degree value screening, and EGFR, PTGS2, SRC and ESR1 were identified as the core targets (Figure 3B). GO and KEGG analyses were performed, and the core targets of EGFR, PTGS2, SRC and ESR1 were input into the Omicshare database to identify the possible mechanism by which acacetin affects sepsis. GO analysis of the CC terms showed that the four core targets were mainly associated with membrane rafts, the membrane microdomain and the membrane region (Figure 3C). GO analysis of BP terms showed that the four core targets were mainly related to the ovulation cycle, steroid hormones and reproductive structure development (Figure 3D). GO analysis of MF terms showed that the four core targets were mainly associated with connexin (Cx) binding, NOS activity regulation, oestrogen receptor binding and protein kinase binding (Figure 3E). KEGG analysis revealed that the four core targets were closely associated with the gap junction, endocrine resistance, oxytocin signalling, proteoglycans in cancer, bladder cancer and human cytomegalovirus infection pathways (Figure 3F). Figure 3G shows the acacetin-sepsis-target-GO-KEGG network.

### Molecular Docking and Experimental Validation of the Binding of Core Acacetin Targets Predicted by Network Pharmacology

Molecular docking was carried out to investigate potential binding modes and interactions between acacetin and its key targets (Figure 4A). For the docking interaction energy score, the targets were ranked as PTGS2 > SRC > ESR1 > EGFR (Table 1). The carbonyl, methoxy and hydroxy groups are thought to be the key groups that bind residues containing H donor moieties at the terminus (Zhao et al., 2019); (Lin et al., 2019); (Duan et al., 2014); (De Savi et al., 2015); (Blobaum et al., 2015) For the specific proteins in the EGFR complex, the residues involved in the formation of H-bonds included ALA 743, THR 790, VAL726, and THR 854. With regard to the SRC docking results, the key residues involved in H-bonds were MET 341, VAL 281 and LEU 393. In the ESR1 complex, THR 347 and MET343 emerged as key residues between the methoxyl group at the 4’ position of acacetin and the target. In the COX-2 (PTGS2) complex, TYR 355 was identified as the only key residue involved in H-bonds. Moreover, known standard or positive molecules of the four targets were subjected to the same simulation (Supplementary Figure S1). The molecular docking results indicated that acacetin can interact with PTGS2, SRC, ESR1 and EGFR to form compact complexes. Src is a prototype of the highly conserved Src family kinase (SFK) proteins, and our results indicated that the expression of p-src in RAW264.7 cells was significantly increased after LPS stimulation and reduced after treatment with acacetin (Figures 4B,C; *p* <0.05).

**TABLE 1:** Interaction of acacetin and hub targets by discovery studio

Targets	AcacetinCDCOKER interaction energy	Positive compareCDOCKER interaction energy
EGFR	37.6692	29.7857
SRC	40.6193	58.7556
PTGS2	49.4521	34.7360
ESR1	37.7927	36.4148

### Experimental Validation of the Acacetin Binding Signalling Pathway Predicted by Network Pharmacology

**FIGURE 5:**
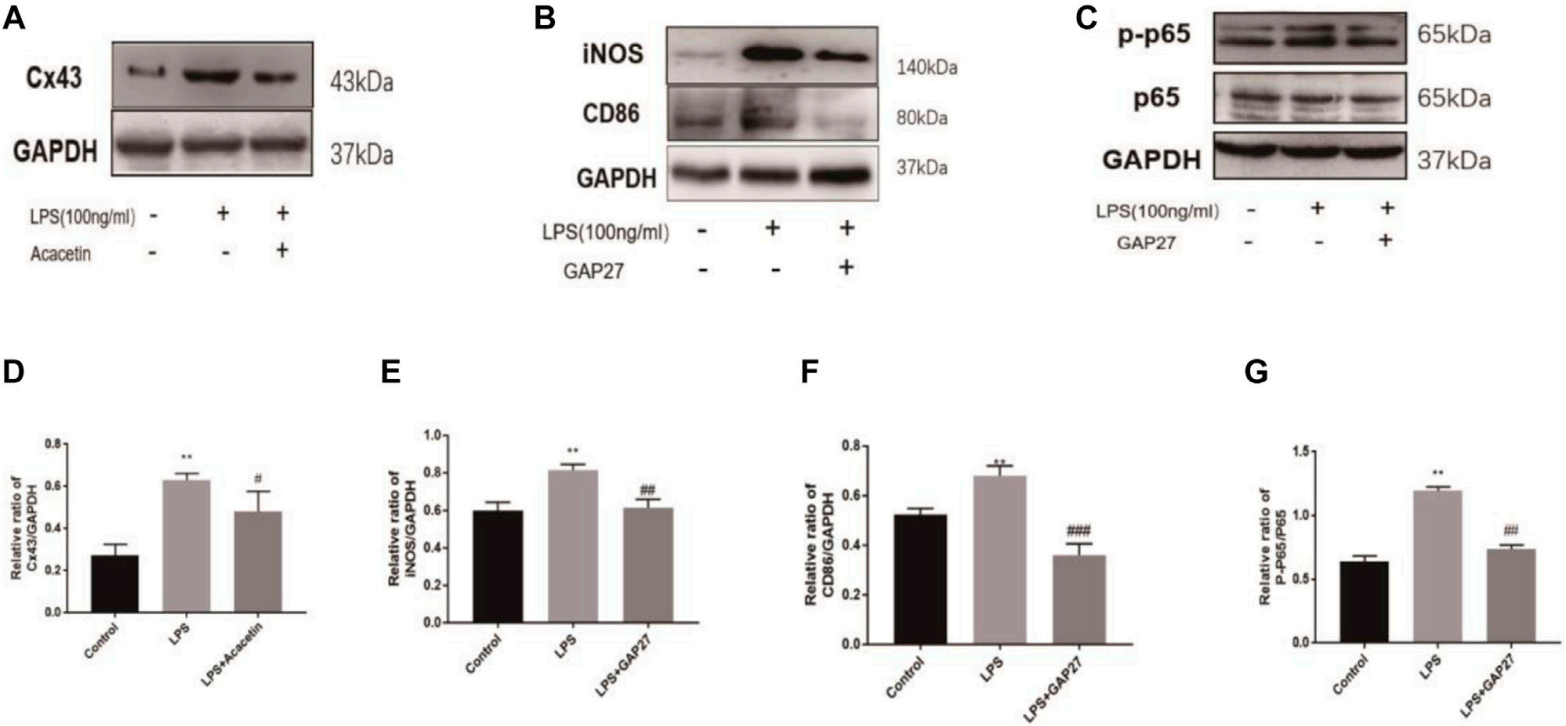
Experimental validation of the acacetin binding signalling pathway predicted by network pharmacology. The expression levels of Cx43, CD86, iNOS, P65 and phosphorylated P65 **(A–G)** were assessed via Western blotting. The data are expressed as the mean ± SEM (*n* = 3). *p < 0.05, **p < 0.01, and ***p < 0.001 compared with the control group; ^#^ *p* < 0.05, ^##^ *p* < 0.01, ^###^ *p* < 0.001 compared with the LPS-stimulated group.

Further network pharmacological analysis and GO and KEGG analyses indicated that gap junctions may be highly involved in the protective effects of acacetin against sepsis. We confirmed the role of gap junctions in the effects of acacetin on LPS-induced M1 polarization in RAW264.7 cells. As shown in Figures 5A,D, the expression levels of Cx43 were significantly increased after LPS stimulation but reduced after treatment with acacetin. As shown in Figures 5B,C,E,F the protein expression levels of CD86, iNOS, p65, and p-p65 were revealed by Western blotting. Notably, p-p65, CD86 and iNOS protein expression was significantly decreased in the Gap27 group compared with the LPS group (*p*<0.05). Together, these results suggest a prospective role for gap junctions in attenuating the effect of acacetin on M1-type macrophages.

## Discussion

Sepsis is a global healthcare issue and continues to be the leading cause of death from infection. In clinical practice, the use of antimicrobial agents to fight infection is key to the treatment of sepsis. Over the past few decades, natural products, such as anti-bacterial and anti-inflammatory drugs, have been used for the herbal treatment and prevention of human diseases (McBride Margaret et al., 2020). Acacetin is a natural flavonoid found in various plants, including *Sparganii rhizoma*, *Sargentodoxa cuneata* and *Patrinia scabiosifolia*. Our results indicated that acacetin improved liver and lung injury by decreasing the levels of IL-1β, IL-6 and TNF-α, and acacetin expedited the reduction in CD86 and iNOS expression in M1-type macrophages. Previous reports have indicated that acacetin has anti-inflammatory effects. For example, acacetin was shown to suppress the LPS-induced upregulation of iNOS and COX-2 in murine macrophages and TPA-induced tumour promotion in mice (Pan et al., 2006). Acacetin was also shown to attenuate mouse ALI induced by endotoxin by augmenting HO-1 activity (Wu et al., 2018) and to block kv1.3 channels and inhibit human T cell activation (Zhao et al., 2014). These results demonstrate that acacetin has anti-inflammatory and antioxidative effects on sepsis, but the underlying molecular mechanism remains unclear.

We herein further investigated the mechanisms underlying the effects of acacetin on sepsis by performing network pharmacology analysis coupled with experimental validation and molecular docking. All potential targets of acacetin and sepsis were methodically obtained, and the top four overlapping targets in the PPI network were EGFR, PTGS2, SRC and ESR1. EGFR prevents the translocation of gut-residing pathogenic and cancer-associated protein kinases and is a key target for late-onset neonatal sepsis and cancer (Knoop et al., 2020; Singh et al., 2021). Our results identified ALA 743, THR790, VAL726, THR854 and ASP855 as the key EGFR residues in the binding pocket. PTGS2, also known as COX-2, has been reported to have an anti-inflammatory effect, and acacetin can reportedly relieve sepsis by inhibiting COX-2 activity (Pan et al., 2006; Wu et al., 2018; Fatemi et al., 2020). Due to the structural specificity of the COX-2 protein, only TYR 355 was identified as the key residue involved in H-bonds. Moreover, ESR1, as the target of the *ShenFuHuang* formulation in a zebrafish model, was associated with septic syndrome in patients with COVID-19 (Liu et al., 2020b). Our results highlighted THR 347 and MET343 as the key residues residing between the methoxyl group at the 4’ position of acacetin and ESR1, which is also consistent with the binding site for the original ligand. Additionally, cellular Src is the prototype of highly conserved SFK proteins. Evidence suggests that siglec-G deficiency ameliorates hyperinflammation and immune collapse in patients with sepsis by regulating Src activation (Li et al., 2019). With regard to the Src docking results, MET 341, VAL 281 and LEU 393 were identified as the key residues of the binding pocket with the original ligand ruxolitinib. Thus, based on its prediction as a core target and its association with macrophages and sepsis, Src deserves to be further studied *in vitro*. Our western blot results suggested that phosphorylated Src plays an important role in the anti-septic activity of acacetin. Together, our results showed that acacetin had a preferential affinity for all four targets, confirming the prediction results obtained by network pharmacology analysis.

GO and KEGG analyses were performed to analyse the interactions among multiple targets and pathways, and the top 20 enriched BPs and signalling pathways were shown to be associated with the therapeutic effects of acacetin on sepsis. Membrane raft, oestrogen receptor activity, and human cytomegalovirus infection are related to the effects of acacetin on sepsis. In a previous study, lipid raft microdomains were shown to be essential components of phagolysosomal macrophage membranes and to play an essential role in antifungal immunity (Schmidt et al., 2020). Moreover, human cytomegalovirus infection was shown to potentially impact immunity (Cox et al., 2021). Thus, our subsequent research will be performed using macrophages. Further network pharmacological analysis indicated that gap junctions may be highly involved in the protective effects of acacetin against sepsis. Cxs are basic components of gap junctions, and Cx43 is a common Cx family member that is widely expressed in immune cells and involved in a variety of immune regulatory processes (Glass et al., 2015). In a previous study, ATP release through Cx43 was reported to be important for inhibiting inflammation and bacterial burden (Balázs et al., 2015). Our results showed that the gap junction selective blocker gap27 could significantly decrease the protein expression of p-p65, CD86 and iNOS. p-p65 is the key target of the classic inflammation-related NF-κB signalling pathway, CD86 is a surface marker of M1-polarized macrophages, and iNOS is a pro-inflammatory molecule associated with the inflammatory process. Our previous research on Cx43 showed that carbenoxolone decreased monocrotaline-induced pulmonary inflammation in rats by decreasing the expression of Cxs in T lymphocytes (Zhang et al., 2020). Angiotensin II induced RAW264.7 macrophage polarization toward the M1 phenotype through the Cx43/NF-κB pathway (Wu et al., 2020). Our findings highlighted gap junctions in macrophages as a new potential target for sepsis, and most importantly, showed that acacetin inhibited the LPS-induced increase in Cx43 expression. Interestingly, the anti-inflammatory effect of acacetin via gap junctions has not been reported. Together, these results suggest that gap junctions attenuate the effects of acacetin on sepsis.

## Conclusion

Our results suggest that acacetin protects against sepsis *via* a mechanism involving multiple targets and pathways and that gap junctions are highly involved in this process.

## Data Availability

The original contributions presented in the study are included in the article/supplementary files, further inquiries can be directed to the corresponding authors.
