# Supplementary figures and images for: A Systematic Study of the Mechanism of Acacetin Against Sepsis Based on Network Pharmacology and Experimental Validation
Source: Front Pharmacol. 2021 Aug 16;12:683645. doi: 10.3389/fphar.2021.683645 (PMC8415621; doi:10.3389/fphar.2021.683645)

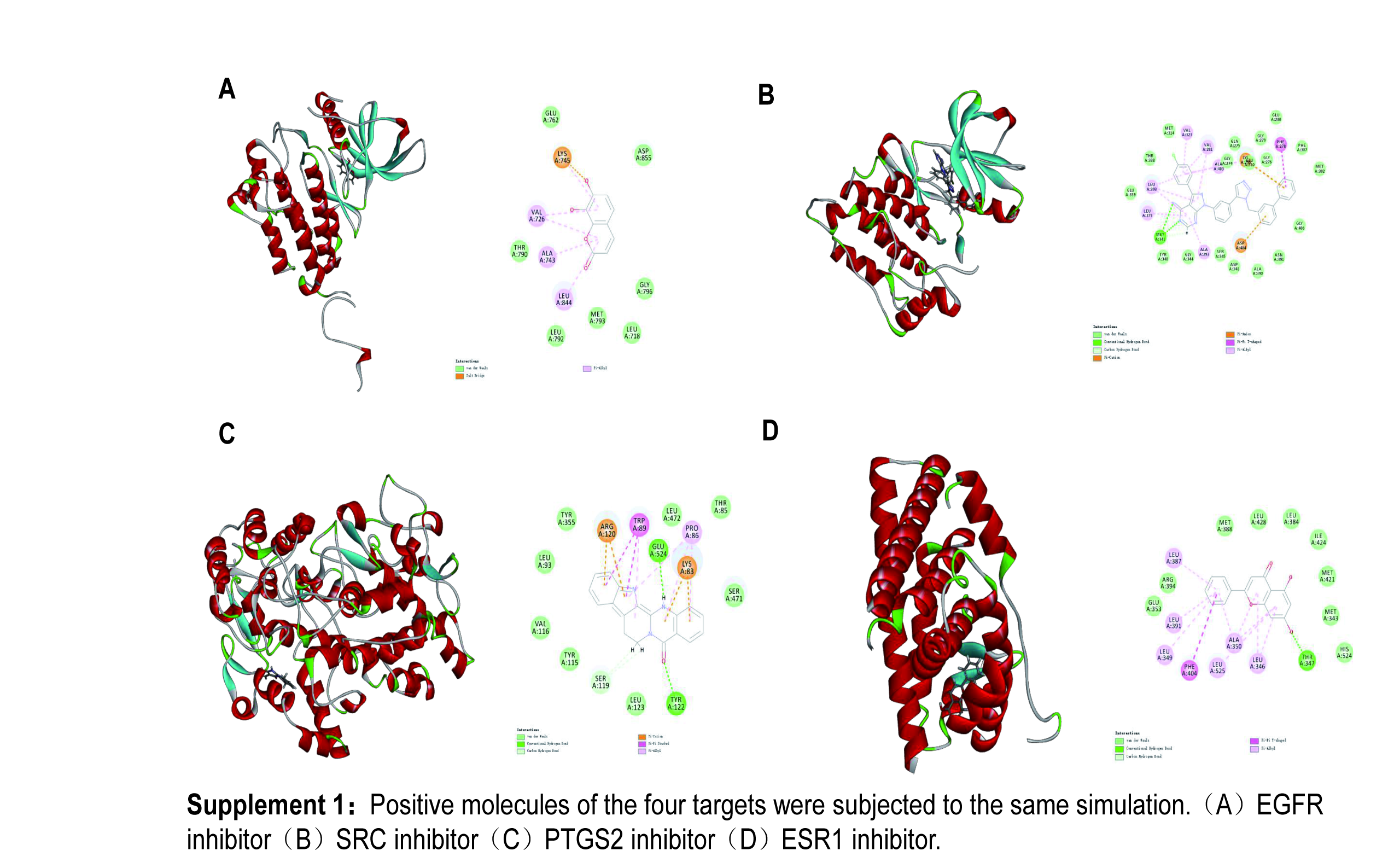

Supplement: Supplementary file 1 [file Image1.tif]
